# Dosimetric Evaluation of the Sensitivity of PAGAT Gel Dosimeters Infused with Clinically Used Gadolinium-Based Contrast Agents

**DOI:** 10.3390/gels11060416

**Published:** 2025-05-30

**Authors:** Melani Fuentealba, Carolina Vallejos, Sergio Díez, Mauricio Santibáñez

**Affiliations:** 1 Departamento de Cs. Físicas, Universidad de La Frontera, Temuco 4811230, Chile; 2 Laboratorio de Radiaciones Ionizantes, Universidad de La Frontera, Temuco 4811230, Chile; 3 Departamento de Fisiología, Universidad de Valencia, 46010 Valencia, Spain; 4 Medical Physics Department, Hospital Clínico Universitario de Valencia, 46010 Valencia, Spain

**Keywords:** gel dosimetry, PAGAT, Gd contrast agent

## Abstract

This study evaluates the impact of gadolinium-based contrast agents (Omniscan, Dotarem, and Gadovist) on the performance of PAGAT gel dosimeters using spectrophotometric analysis. Dosimeters were infused with gadolinium at concentrations ranging from 0 to 40 mg/mL and irradiated with a 6 MV photon beam over a dose range of 0–15 Gy. Regarding dosimeter behavior, Dotarem exhibited an enhancement in optical density prior to irradiation due to polymerization reactions between the dosimeter and the contrast agent starting at 10 mg/mL, which compromised optical readings above 20 mg/mL. Omniscan consistently showed 37.7% lower sensitivity than standard PAGAT across all concentrations and dose levels. Conversely, Gadovist enhanced sensitivity by up to 50% at 20 mg/mL, with additional gains at higher concentrations, although accompanied by saturation at lower dose levels. Radiological analysis showed that all tested concentrations maintained mass energy–absorption coefficient differences below 1% and water-equivalence in effective atomic number within 5% at 6 MV. These findings underscore the importance of selecting an appropriate contrast agent to enhance gel dosimeter sensitivity, particularly in low-dose regions where measurement uncertainty increases. Additionally, gadolinium-infused PAGAT gels show strong potential for assessing dose enhancement phenomena. Their sensitivity, threshold behavior, and radiological properties suggest they may be suitable for applications in dose enhancement dosimetry as well as conventional clinical settings.

## 1. Introduction

Gel dosimetry is characterized, unlike conventional dosimeters commonly used, by its ability to provide a spatial dose distribution, which can be processed using imaging techniques such as MRI [1]. One of the main disadvantages of this type of dosimeter in its early stages was that it required anoxic or hypoxic preparations since oxygen inhibits the radiation-induced polymerization reaction [2]. This limitation was addressed with the creation of the dosimeter known as MAGIC, in which two of its components—ascorbic acid and copper—form a complex with oxygen, which, along with radiolysis of water, serves as a free radical source for the initiation of the polymerization of methacrylic acid [3]. Later, other antioxidants, such as THPC, were introduced and added to existing acrylamide-based polymer gel dosimeters, leading to the development of the current PAGAT dosimeters [4], which can be prepared under normoxic conditions.

However, the use of THPC can also affect the response of the dosimeter. Antioxidant molecules that do not react with oxygen may also react with water radicals, polymer radicals, or both [5]. At high concentrations, THPC can decrease the response of the dosimeter (evaluated by MRI and spectrophotometry), not by altering the polymerization itself, but by changing the structure of the resulting polymer, modifying the light scattering properties and/or the bonding behavior between water molecules and the polymer micelles [6]. Jirasek et al. reported that THPC can react with the amine chains of gelatin prior to irradiation, increasing coagulation and cross-linking in the gelatin matrix, suggesting a chemical alteration that reduces dose sensitivity [7]. So far, no reaction between THPC and acrylamide and/or bis-acrylamide has been observed, though it cannot be ruled out. However, raising the pH to alkaline levels has shown a reaction between THPC, acrylamide, and bis-acrylamide [7,8].

Beyond the effects of THPC, various physical and chemical factors can alter gel dosimeter composition and behavior pre- and post-irradiation. Djezzar notes that pH and temperature influence cross-linking, with high temperatures (>50 °C) promoting chain breakage. Polymerization also correlates with radiation dose, and UV light can initiate polymerization via water photolysis, generating reactive radicals. [9].

Regarding dosimeter composition, the type and nature of the solvent are critical. A solvent with low solubility capacity may impair polymerization processes, increasing molecular weight variability. For example, water generates more radicals than polymers or monomers, increasing polymerization events [9]. The gel matrix structure, affected by mesh size, pH, temperature, concentration, and additives, influences species diffusion. These factors can alter matrix integrity, inhibit polymerization, affect solubility, or enhance performance. Additionally, matrix preparation and solvent/additive interactions determine initial properties and may cause premature polymerization [5]. Impurities in water or aged components can consume radicals, impacting modeling reliability if unaccounted for. [5,9].

Some studies also indicate that metal nanoparticles can promote chain reactions. The radiation-induced production of radicals is enhanced by the presence of high-Z nanoparticles, although this may also increase the likelihood of radical recombination. Certain substances, such as silver nitrate, act as radiation-sensitive materials that induce the synthesis of nanoparticles (AgNPs) [5,10].

Currently, PAGAT gel dosimeters have been extensively studied and used for clinical dose evaluation in various complex radiotherapy techniques such as Intensity-Modulated Radiation Therapy (IMRT) [11], Stereotactic Radiosurgery (SRS) [12,13,14], brachytherapy (BQT) [15,16], proton therapy [17,18,19], among others. Among the qualities that make this dosimeter useful for clinical dosimetric evaluation is its linear dose response within the clinical dose range. Since the amount of polymerization produced is directly proportional to the absorbed dose [20], its radiological equivalence to water—within 1% for energies above 100 kV and within 5% for energies between 10 and 100 kV [21]; negligible energy dependence for 6–21 MeV electron beams and 1.25–18 MV photon beams [22]; dose rate independence for megavoltage beams between 25 and 400 cGy/min [23]; and its ability to provide three-dimensional dose distribution evaluation, as it maintains spatial correlation between the site of radiation incidence and the hydrogel matrix.

Another advantage of this type of gel dosimeter lies in its ability to be infused with additional materials, which has been explored primarily for two purposes. The first is to enhance the dosimeter sensitivity at low radiation doses [24,25,26], a strategy applied to both diagnostic and therapeutic beams. Several materials have been studied as radiosensitizers in this context. For example, formaldehyde increased the sensitivity of the MAGIC gel dosimeter by 10.5% when irradiated with a ^60^Co beam [25]. Inorganic salts like MgCl_2_ showed a 2.8-fold increase in sensitivity after exposure to a 6 MV beam [26]. Organic molecules such as glycerol and isopropanol, used in NIPAM dosimeters, yielded sensitivity increments of approximately 50% at a dose of 20 Gy when using a 6 MV beam with 30% glycerol content [27] and up to 51% with isopropanol [28]. Additionally, iodine-based contrast agents incorporated into NIPAM and MAGAT polymer dosimeters—when irradiated with X-rays from a CT scanner—achieved sensitivity increases of 3.35 ± 0.6 for NIPAM and 1.36 ± 0.3 for MAGAT [16].

Secondly, the infusion of gel dosimeters has focused on the study of the dose enhancement phenomenon, which is produced due to the infusion of high atomic number materials [29]. PAGAT gel, which has a water equivalence of 1% for energies over 100 kV and 5% for energies under 100 kV, allows the evaluation of the dosimetric effect of these agents as dose enhancers in the body. When irradiated near their K-edge, these materials emit localized photoelectrons and Auger electrons within the dosimeter, a phenomenon undetectable by conventional dosimeters like ionization chambers and radiochromic films [29], creating the need for the development of dosimeters that incorporate the high-Z element within their sensitive volume. For this purpose, several high-Z nanoparticles have been studied. For example, gold nanoparticles produce dose enhancements in the MAGICA dosimeter of up to 1.12 times [30]; silver nanoparticles infused in nMAG and NIPAM dosimeters produce enhancement factors of 1.43 and 2.4, respectively, for 120 kVp beams [31]; silver nitrate with glycerol in a PAG dosimeter increased the dose by 30% compared to a dosimeter infused only with silver nitrate [32]; Bismuth-infused nPAG dosimeters resulted in a 16.35% dose enhancement for ^192^Ir beams [33]; platinum nanoparticles increased the dose by 27.1% when irradiated with a ^60^Co beam [34]; and BiGdO_3_ produced an enhancement factor of 2.3 for 160 kVp beams [35].

While nanoparticles are commonly used for dose enhancement, contrast agents are more affordable, accessible, and have the lowest toxicity to the body. Contrast agents like iodine have shown effectiveness, for example, a 1.82-fold increase in PAG dosimeters at 12 mg/mL with 40 keV beams [36]. Gadolinium-based compounds have also been infused into PAGAT gels and have demonstrated enhancements, yielding both experimental and Monte Carlo simulation results at various contrast agent concentrations. These showed increases of up to 2.45 times with a 57 kVp beam quality [29] and enhancements of 1.61 times with a 160 kVp beam [37].

In this study, the behavior of PAGAT gel dosimeters infused with three types of clinical gadolinium contrast agents from different generations (Omniscan, Gadovist, and Dotarem) and at different concentrations will be evaluated using optical spectrophotometry.

As previously mentioned, introducing other compounds could potentially modify the properties of the dosimeter. This would imply that, despite having the same radiological properties due to the same concentration of a high atomic number element, the chemical nature of each compound could modify the dose–response curve, thus affecting dosimetric interpretation. The need to deconvolute the physical dose enhancement process and the chemical effects of the contrast agents on the dosimeter requires evaluation with a beam quality that minimizes dose enhancement generation by gadolinium (6 MV WFF beam).

The aim is to produce a dosimeter capable of evaluating dose enhancement, which in the case of gadolinium is strongly influenced by kV energies such as those produced in unflattened beams (FFF), BQT, and, more recently, electronic BQT. Given that increments in dose enhancement are localized and relatively low, it is necessary to improve dosimeter properties such as sensitivity and dose threshold to lower dose ranges, which would benefit the evaluation of dose enhancement. These improvements would also be potentially useful in conventional dosimetry as patient-specific RT dosimetry, allowing for the evaluation of organs at risk and dose gradients with reduced uncertainty, given its higher sensitivity.

## 2. Results and Discussion

### 2.1. Gd-PAGAT Elaboration Viability

Regarding the behavior of PAGAT dosimeters infused with different contrast agents, it was observed that, in the specific case of Dotarem, a polymerization reaction occurred within the dosimeter one hour after preparation, particularly at concentrations of 10 mg/mL and 20 mg/mL. Moreover, the degree of polymerization was directly proportional to the concentration of the contrast agent.

**Figure 1:**
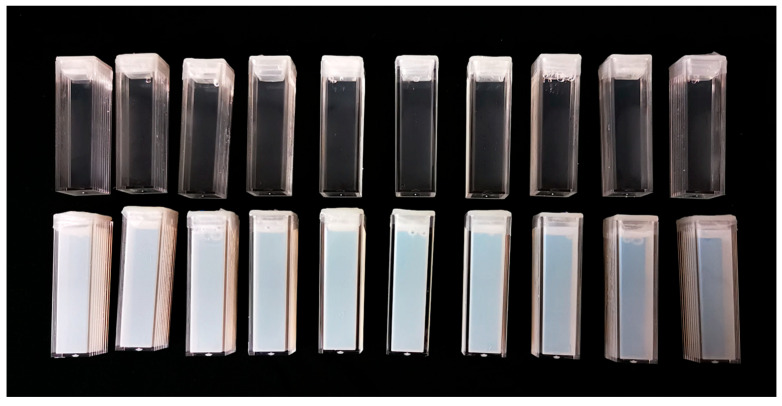
PAGAT dosimeters (**top**) and PAGAT dosimeters infused with Dotarem at 20 mg/mL Gd concentration (**bottom**), showing increased opacity due to the reaction between the contrast agent and the PAGAT formulation.

The onset of the reaction prior to full polymerization is visibly evident in Figure 1 and quantitatively supported by the pre-irradiation absorbance values shown in Table 1. While the absorbance values for PAGAT and the lowest Dotarem concentration were statistically equivalent, a significant enhancement was observed at concentrations of 10 mg/mL and 20 mg/mL, with minimum enhancement factors of 1.24 and 19.17, respectively. Additionally, greater variability was noted at higher concentrations, indicating increased deviation between measurements.

However, despite its early polymerization, after irradiation, it maintained a dose-dependent increase in response in both cases. Particularly at the 20 mg/mL concentration, absorbance values are so high that they exceed the spectrophotometer upper reading limit starting at 4 Gy.

Regarding the dosimeters infused with Omniscan and Gadovist, as the contrast agent concentration increases—and considering that these agents have lower absorbance than PAGAT—a decrease in the dosimeter absorbance would be expected. This trend is indeed observed with Gadovist, where a progressive reduction in absorbance occurs as concentration increases. However, this behavior is not replicated at the 20 mg/mL concentration of Omniscan, where an increase in absorbance is observed compared to PAGAT. This suggests that the infused dosimeter undergoes a reaction that does not significantly affect the initial absorbance, yielding values comparable to those obtained with standard PAGAT.

### 2.2. Dosimetric Evaluation According to Contrast Agent

When evaluating both by contrast agent and concentration, significant differences were observed in comparison to conventional PAGAT, particularly in dosimeter sensitivity and dose–response threshold. These results are summarized in Table 2 at the end of the section.

**Figure 2:**
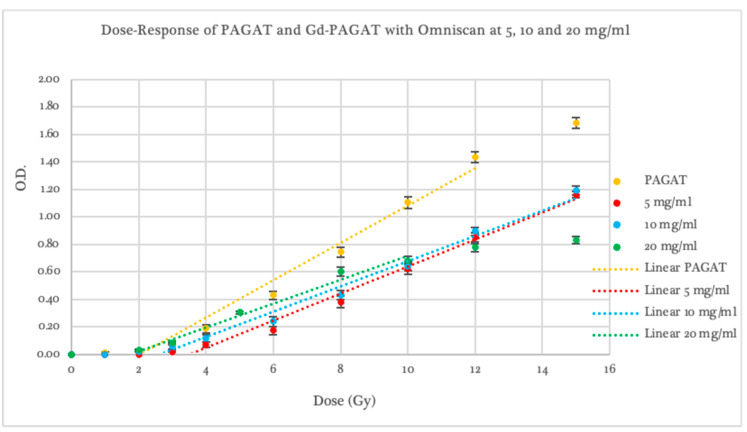
Dose–Response curve of PAGAT and PAGAT infused with the contrast agent Omniscan at concentrations of 5, 10, and 20 mg/mL, including trend lines in the linear dose range.

Using PAGAT as the reference dosimeter, all concentrations prepared with the contrast agent Omniscan (see Figure 2) exhibited a lower response to radiation within the evaluated dose range. The threshold dose was 3.50 ± 0.53 Gy for the 5 mg/mL concentration, 3.35 ± 0.31 Gy for 10 mg/mL, and 1.75 ± 0.33 Gy for 20 mg/mL. Regarding dosimeter sensitivity, an average reduction of 37.7% in the slope of the linear dose–response curve was observed across all three Omniscan concentrations, with slopes ranging from 0.087 to 0.101, compared to 0.153 for standard PAGAT.

Furthermore, at the 20 mg/mL concentration, curve saturation occurred beyond 8 Gy, a behavior not observed at lower concentrations, which maintained linearity across the entire dose range. Overall, while Omniscan-infused dosimeters did not provide a significant improvement in threshold or sensitivity compared to PAGAT, the 5 and 10 mg/mL concentrations did maintain linearity in response without saturation, unlike standard PAGAT.

**Figure 3:**
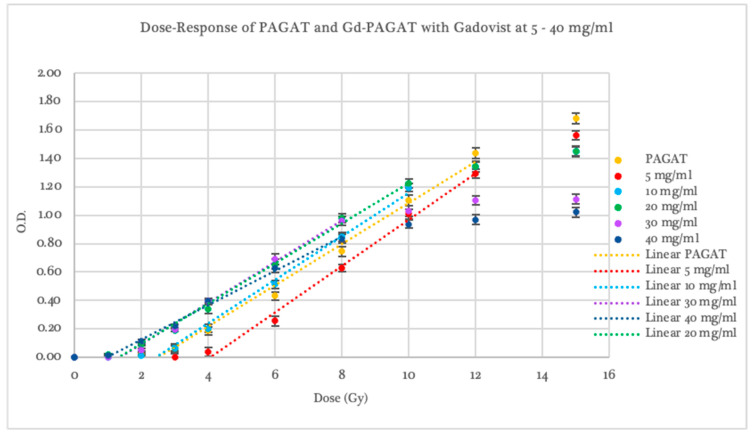
Dose–Response curve of PAGAT and PAGAT infused with the contrast agent Gadovist at concentrations of 5, 10, 20, 30, and 40 mg/mL, including trend lines in the linear dose range.

Gadovist exhibited greater sensitivity than Omniscan, with variations depending on concentration, as shown in Figure 3. At a concentration of 5 mg/mL, the dosimeter showed lower response than PAGAT across the entire evaluated dose range but higher sensitivity than Omniscan at the same concentration, with a dose threshold of 4.04 ± 0.46 Gy. A linear response was observed between 4 and 12 Gy, with an R^2^ value of 0.994.

The 10 mg/mL concentration showed a dose threshold beginning at 2.72 ± 0.07 Gy, with higher optical density readings than PAGAT in the 4–10 Gy range. It yielded a linear dose response between 2 and 10 Gy, with an R^2^ of 0.991. Concentrations of 20, 30, and 40 mg/mL demonstrated an improved response threshold, starting at 1.55 ± 0.12 Gy, 1.60 ± 0.11 Gy, and 1.01 ± 0.17 Gy, respectively (compared to the 2.46 ± 0.13 Gy threshold of conventional PAGAT). Optical density readings were notably higher at lower dose ranges: 2–10 Gy for the 20 mg/mL concentration and 1–8 Gy for 30 and 40 mg/mL. Beyond these ranges, signal saturation began at 8 and 10 Gy. In contrast, the 5 mg/mL concentration is saturated at the same dose (12 Gy) as standard PAGAT.

In terms of sensitivity, dosimeters infused with 5 and 10 mg/mL of Gadovist exhibited slopes that were 6.5% and 5.9% steeper, respectively, compared to conventional PAGAT. The 30 mg/mL concentration showed the same slope as PAGAT, while the 20 and 40 mg/mL concentrations presented shallower slopes, with values 4.1% and 20.3% lower, respectively. These results suggest that as the concentration of Gadovist increases, optical density values rise predominantly at lower doses, up to approximately 4 Gy. Within this range, higher sensitivity is observed, followed by a rapid decline as the response curve begins to saturate. The concentrations that best balance dose threshold and optical density response at low doses while avoiding curve saturation at higher doses are 10 and 20 mg/mL, with the 20 mg/mL concentration standing out for its superior performance in the low-dose region.

As previously mentioned, the contrast agent Dotarem, unlike the other compounds used, showed a visible change in the opacity of the dosimeter due to polymerizations occurring before irradiation. These reactions were proportional to the concentration of the agent in the dosimeter and resulted in higher absorbance values in spectrophotometric readings compared to the other samples. This effect became apparent starting at a concentration of 10 mg/mL. A possible explanation for this behavior may lie in the chemical nature of Dotarem, which is the only contrast agent used with a monocyclic ionic structure. This could imply that, when dissolved in water, it dissociates fully or partially into ions that have the potential to modify the polymerization process—either by altering the distribution of free radicals or by interacting with other components of the gel, such as THPC. It has been reported that THPC molecules that do not react with oxygen can also react with free radicals from water and/or polymer radicals [5]. Additionally, it has been reported that in aqueous solutions, the ligand DOTA, present in this compound, can react with water-derived radicals [38].

**Figure 4:**
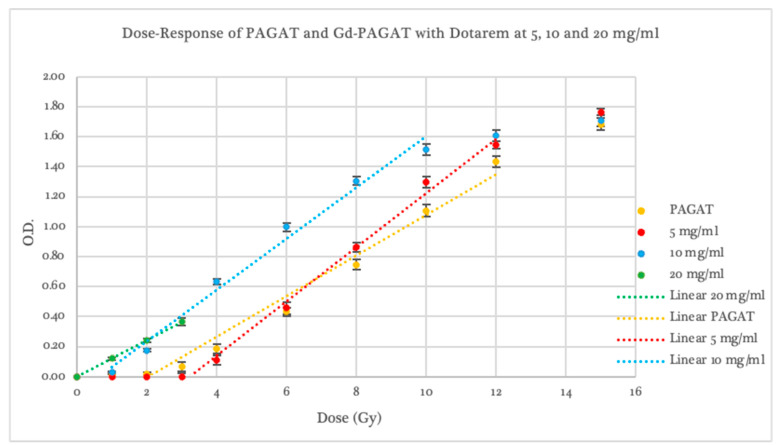
Dose–Response curve of PAGAT and PAGAT infused with the contrast agent Dotarem at concentrations of 5, 10, and 20 mg/mL, including trend lines in the linear dose range.

At the 20 mg/mL concentration, the pre-irradiation polymerization significantly affected the dosimeter sensitivity measurements, causing spectrophotometric readings to exceed the measurable range beyond 4 Gy, as shown in Figure 4. In this case, a linear response was observed between 1 and 3 Gy, with optical density values higher than those of all other evaluated dosimeters. However, the slope was 20.9% shallower than that of conventional PAGAT.

In terms of irradiation response, the 5 mg/mL Dotarem concentration exhibited a dose threshold of 3.40 ± 0.42 Gy, with optical density values exceeding those of PAGAT in the 6–15 Gy range. Like conventional PAGAT, saturation occurred at 12 Gy. This concentration showed a sensitivity 21.6% higher than that of the reference dosimeter.

For the 10 mg/mL concentration, although pre-irradiation polymerization was observed, the post-irradiation dose–response curve demonstrated a threshold beginning at 0.64 ± 0.32 Gy, with consistently higher optical density values than PAGAT across the entire evaluated dose range. A linear response was observed between 1 and 10 Gy, with a slope 11.8% steeper than that of the conventional formulation, as shown in Figure 4.

Therefore, while increasing the concentration of Dotarem may enhance the dosimetric response, the chemical composition of this contrast agent leads to pre-irradiation polymerization effects that compromise stability. As such, it would not be suitable for use with this specific gel dosimeter formulation.

### 2.3. Dosimetric Evaluation According to Gd Concentration

The concentration-based analysis of Gd was conducted using 5, 10, and 20 mg/mL, as only these concentrations were common across all three contrast agents evaluated.

**Figure 5:**
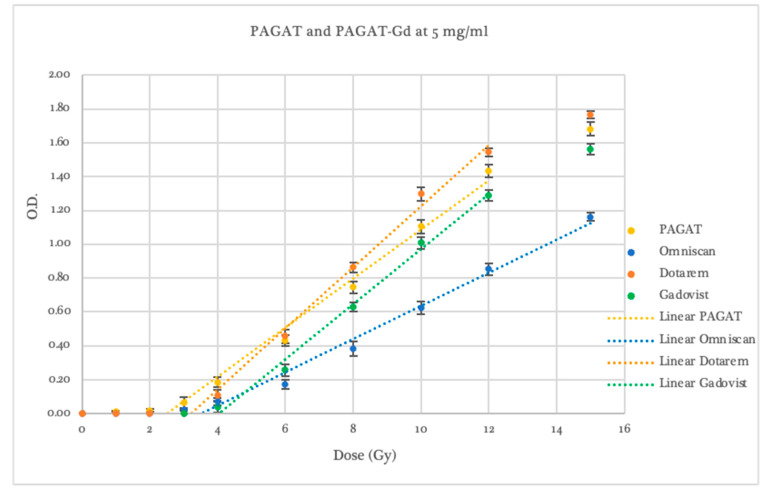
Dose–Response curves of PAGAT and PAGAT infused with Dotarem, Omniscan, and Gadovist at a concentration of 5 mg/mL, including trend lines in the linear dose range.

At the 5 mg/mL concentration, all contrast agents exhibited lower responses than conventional PAGAT at low doses. From 6 Gy onwards, Dotarem demonstrated a higher response and greater sensitivity (statistically significant, *p* < 0.05), as previously noted, with a dose–response curve and saturation behavior similar to that of standard PAGAT, as shown in Figure 5. However, at this concentration, none of the contrast agents showed a significantly favorable difference compared to PAGAT.

**Figure 6:**
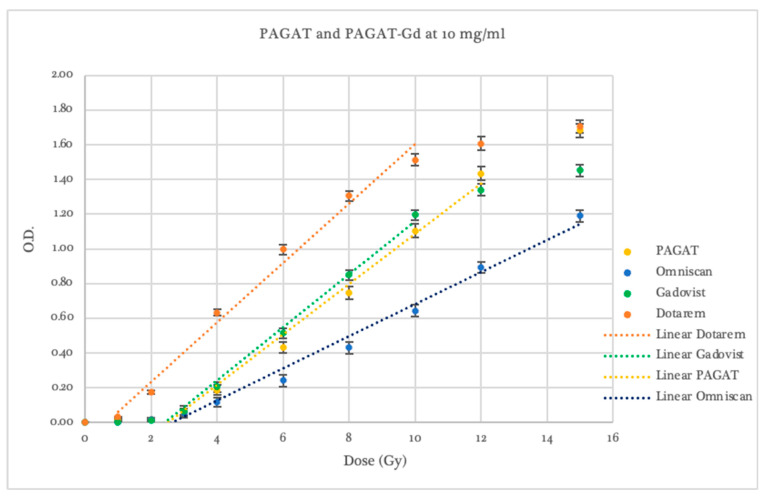
Dose–Response curves of PAGAT and PAGAT infused with Dotarem, Omniscan, and Gadovist at a concentration of 10 mg/mL, including trend lines in the linear dose range.

At a concentration of 10 mg/mL (see Figure 6), with the exception of Omniscan, which demonstrated inferior performance (slope and threshold statistically lower than PAGAT, *p* < 0.05), both Gadovist and Dotarem showed a higher response compared to PAGAT.

Gadovist exhibited a 5.9% steeper slope than PAGAT and maintained linear behavior in the 2–10 Gy dose range; however, its response curve saturated beyond 10 Gy. Dotarem, on the other hand, displayed significantly improved performance in terms of dose threshold, sensitivity, and optical density values (statistically significant, *p* < 0.05). Nevertheless, pre-irradiation polymerization led to response instability that was dependent on the time elapsed between dosimeter preparation and irradiation. For this reason, the use of Dotarem at concentrations above 5 mg/mL is not recommended when using spectrophotometric readings.

**Figure 7:**
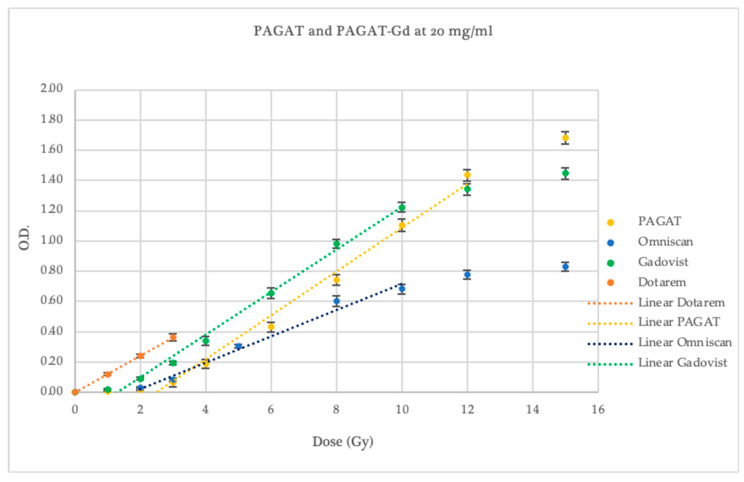
Dose–Response curves of PAGAT and PAGAT infused with Dotarem, Omniscan, and Gadovist at a concentration of 20 mg/mL, including trend lines in the linear dose range.

At a concentration of 20 mg/mL (see Figure 7), the three contrast agents exhibited distinctive behaviors. Omniscan showed a response similar to that of conventional PAGAT at low doses, followed by saturation beginning at 8 Gy, offering no significant advantage over the reference dosimeter.

Dotarem, despite the previously mentioned issues with pre-irradiation polymerization and out-of-range absorbance values, displayed linear behavior within the dose range it was able to respond to, starting from 0.01 ± 0.01 Gy (statistically significant, *p* < 0.05). At 1 Gy, the optical density value for Dotarem was equivalent to that of 3.7 Gy in the PAGAT scale. Although curve behavior beyond this point could not be evaluated due to saturation at the spectrophotometer’s maximum absorbance limit, the slope trend suggests lower dose uncertainty in the 1–3 Gy range.

Gadovist demonstrated the best performance among the three agents, with optical density values exceeding those of PAGAT up to approximately 11 Gy. Beyond 10 Gy, the response curve began to saturate. While it exhibited a slope 4.1% shallower than PAGAT, its response at low doses was markedly superior; for instance, at 2 Gy, its optical density was equivalent to that of 3.5 Gy in conventional PAGAT.

### 2.4. Radiological and Dosimetric Properties

Table 3 shows the radiological characteristics of each compound and gadolinium concentration used for a range of effective energies commonly employed in radiotherapy treatments with MV beams. As observed, the μ_en_/ρ values of all evaluated compounds differ by less than 1% compared to conventional PAGAT.

Regarding water equivalence, assessed through Z_PEAeff_ values, it can be noted that it varies depending on the energy considered. For the effective energy of 1.60–1.75 MeV, the percentage difference compared to PAGAT was below 5% for all compounds at concentrations up to 30 mg/mL. For energy of 3.8 MeV, values remained below 5% up to 10 mg/mL for Omniscan and Dotarem and up to 20 mg/mL for Gadovist. At an effective energy of 5 MeV, no water equivalence was observed for any compound. However, the percentage difference from water does not exceed 10%.

## 3. Conclusions

The choice of the high-Z agent used to infuse PAGAT-type gel dosimeters during the fabrication of dosimeters intended for dose enhancement quantification plays a crucial role in improving their dosimetric properties in terms of sensitivity and threshold. This approach aims to address an aspect that, to date, has posed significant challenges for experimental determination.

Furthermore, considering that these optimizations in the dosimeter do not involve the dose enhancement phenomenon due to the radiation beam quality used, this represents a significant opportunity to improve dose assessment in complex radiation therapy treatments.

Regarding the radiological equivalence of the dosimeter infused with the Gd agent compared to water, it was observed that the dosimeters, for all evaluated concentrations and contrast agent types, maintain a (μ_en_/ρ) value similar to that of water, with a difference of less than 1%. For nominal energies of 6 MV, the difference remains below 5% compared to PAGAT up to a concentration of 30 mg/mL. For 18 MV beams, this difference increases, reaching up to 10% for all concentrations. However, even at concentrations or energies outside of water equivalence, they could eventually be used under similar considerations as other rare-earth-doped dosimeters, such as TLD-400, which require appropriate correction factors to compensate for medium discrepancies.

Among the evaluated contrast agents, Gadovist stands out due to its higher molar concentration (1 mmol/mL), which enables the formulation of higher-concentration dosimeters using smaller volumes of contrast agents without altering the original component ratios. In terms of optimizing dosimeter sensitivity, lower concentrations of Gadovist were found to increase the slope of the dose–response curve, preserving a useful linear range similar to that of conventional PAGAT. However, as the Gd concentration increases, the usable range narrows, and the curve begins to saturate at intermediate doses (8–10 Gy). At higher concentrations, the dosimeter becomes more sensitive at lower doses.

The concentration that offers the best balance between enhanced sensitivity and preservation of a usable linear range is 20 mg/mL. At this level, the dosimeter maintains a linear response between 1 and 10 Gy while providing a 50% enhancement in optical density in the 1–4 Gy range compared to conventional PAGAT.

The differences observed among the contrast agents suggest that their chemical structure influences their effects on polymerization. Of the three agents studied, only Dotarem is ionic and exhibits non-radiation-induced polymerization reactions prior to irradiation. This implies that, when diluted in water (or under the gel conditions), it dissociates fully or partially into ions, which may alter the polymerization process either by modifying the distribution of free radicals or through interactions with other gel components, such as THPC, thereby initiating the polymerization reaction.

Gadovist, on the other hand, is non-ionic and macrocyclic, resulting in high structural stability. Being electrically neutral, it is less likely to interact with any component of the PAGAT gel, meaning that the release of Gd^3+^ would be purely radiation-induced, promoting polymerization only upon exposure to ionizing radiation.

In the case of Omniscan, which is a linear and non-ionic compound, it is the least stable among the contrast agents in both thermodynamic and kinetic terms, with a higher likelihood of decomposition. Additionally, this type of compound typically includes a coordinated water molecule acting as a ligand, which enters and exits the Gd^3+^ coordination sphere—a process known as water exchange. In the case of gadodiamide, the weaker bonding results in a slower exchange rate compared to the other contrast agents, which could affect polymerization by reducing the availability of Gd^3+^ to interact with the gel environment. However, this does not prevent a portion of Gd^3+^ from potentially being released and interfering with the polymerization process.

## 4. Materials and Methods

### 4.1. Gd-PAGAT Infused Dosimeter

The preparation of the PAGAT polymer gel dosimeters was performed based on the methodology published by Venning et al., 2004 [39], maintaining the same monomer ratios and a fixed concentration of 10 mM THPC for all proposed cases in order to evaluate only the contrast agent as a variable. The non-doped PAGAT formulation was kept at the typical concentration of 5% *w*/*w* gelatin, 3% *w*/*w* acrylamide, 3% *w*/*w* BIS, approximately 89% *w*/*w* double-distilled water, and 10 mM THPC.

The doped dosimeters were infused with three different Gd-based contrast agents: Omniscan (GE HealthCare, Chicago, IL, USA), Dotarem (Guerbert, Villepinte, France), and Gadovist (Bayer, Leverkusen, Germany), which are described later. For each contrast agent, different concentrations were prepared. In the case of Omniscan and Dotarem, which both have a molar concentration of 0.5 mmol/mL, concentrations of 5 mg/mL, 10 mg/mL, and 20 mg/mL were evaluated. For Gadovist, which has a higher molar concentration of 1.0 mmol/mL, additional higher concentrations of 30 mg/mL and 40 mg/mL were also evaluated.

Two independent measurements were made for each condition, where each of the 10 dose points evaluated was measured with 3 dosimeters.

In the preparation of the infused PAGAT dosimeters, the Gd-based compounds were added during the final stage along with the antioxidant THPC, replacing between 5.1% and 47.1% of the total water content (depending on the concentration of Gd added). This substitution is feasible due to the inherent water content in the contrast agents, which is approximately 590 mg/mL, allowing for proper dilution of the THPC. The proportions of ingredients for each contrast agent concentration used are detailed in Table 4.

As shown in Table 4, in the case of the higher gadolinium concentrations—specifically 20 mg/mL for Omniscan and Dotarem, and 30 mg/mL and 40 mg/mL for Gadovist—the distribution of reagents differs from that of standard PAGAT due to the amount of contrast agent that needs to be incorporated (ranging from 22.0% to 29.7%). This is because simply adjusting the water content in the original formulation was not sufficient; as such, a reduction alone would prevent the proper dissolution of gelatin, BIS, and acrylamide. Without adjusting other reagents, it would have been necessary to add the contrast agent at the beginning of the process, which would increase the likelihood of premature reactions with the other components.

After preparation, both the Gd-PAGAT and conventional PAGAT dosimeters were stored in polystyrene spectrophotometry vials measuring 4.5 × 1.0 × 1.0 cm^3^ and kept refrigerated at 4 °C for 24 h to ensure complete gelation.

#### Gd Contrast Agent

Three types of paramagnetic MRI contrast agents based on Gd with chelating complexes were used: Omniscan (Gd-DTPA-BMA), whose active ingredient is gadodiamide, is a linear, non-ionic agent and, among this class of contrast agents, the least molecularly stable. Gadovist (Gd-DO3A), whose active ingredient is gadobutrol, is a macrocyclic, non-ionic compound, and Dotarem (Gd-DOTA), with gadoterate meglumine as its active ingredient, is a macrocyclic, ionic compound, both of which are considered highly stable [40].

The mentioned stability refers to the chelating agent’s ability to remain bound to the gadolinium ion. Unlike linear chelates, macrocyclic chelates require the simultaneous or near-simultaneous breaking of multiple bonds between Gd and nitrogen to release the ion, which is less likely to occur. This stability can be affected by temperature and pH. Elevated temperatures above 40–50 °C and acidic pH conditions facilitate the dissociation of the gadolinium ion from the chelating agent [41]. This is important to consider, given that the prepared PAGAT dosimeter has a pH between 5.5 and 6.5.

### 4.2. Irradiation System

Irradiation was performed using a clinical 6 MV linear accelerator (UNIQUE, Varian Medical Systems, Palo Alto, CA, USA). This beam quality was selected due to the negligible dose enhancement in the presence of gadolinium (absorption edge at 50.2 keV), ensuring that any observed variations in dosimeter response would be attributable to differences in chemical composition rather than radiation interaction effects. The equipment used was dosimetrically calibrated based on the IAEA TRS-398 Code of Practice. Measurements were made with a PTW Farmer-type chamber, model 30013, which has a sensitive volume of 0.6 cc.

**Figure 8:**
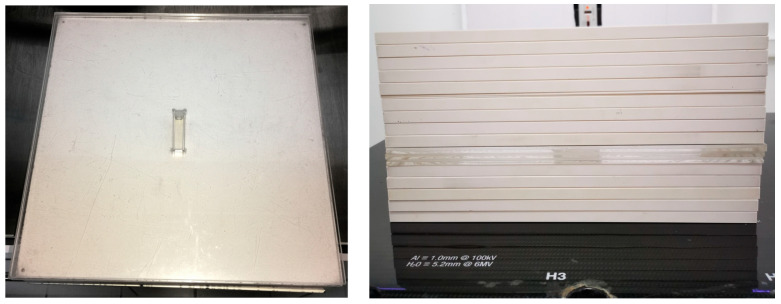
On the (**left**), a diagram of the irradiation setup is shown, displaying an acrylic slab with an insert for the spectrophotometry vials, while on the (**right**), the setup is shown with vials at a depth of 10 cm.

The dosimeters were placed in polystyrene spectrophotometry vials and irradiated under reference conditions based on the TRS-398 practice guideline. The vials were irradiated in a solid water phantom of 30 × 30 × 15 cm^3^, which included a PMMA slab of 30 × 30 × 1.5 cm^3^ machined by CNC for perfect vial fitting in the phantom (as shown in Figure 8). Additionally, 5 g/cm^2^ was considered below the vial to compensate for backscatter. An SSD of 100 cm, a field size of 10 × 10 cm^2^, and an irradiation depth of 10 cm were used. The irradiation was performed with two opposing parallel treatment fields, AP and PA (with vial position change in the phantom), to ensure irradiation homogeneity. The dose rate delivered to the dosimeter was 3.3 cGy/s with a pulse frequency of 300 MU/min. The dose range used to irradiate the dosimeters was 1–15 Gy, leaving a control sample unirradiated.

### 4.3. Dosimeter Processing

Sample processing was carried out using optical spectrophotometry with a QUIMIS Q-798UVN spectrophotometer, featuring a spectral bandwidth of 5 nm and a wavelength range of 195–1000 nm, with a precision of ±2 nm. Spectrophotometry vials were analyzed at a wavelength of 530 nm in absorbance mode, 24 h after preparation (prior to irradiation), and, again, 24 h post-irradiation.

Before measurement, the vials were kept at room temperature for 1 h to minimize fluctuations in absorbance readings caused by condensation on the vial walls due to temperature gradients. Each dosimeter was measured in triplicate. Optical density (OD) was calculated as the average change in optical absorbance after irradiation relative to pre-irradiation values, along with the corresponding standard deviation.

The threshold dose and sensitivity of the dosimeters were evaluated, with sensitivity determined from the slope of the dose–response curve.

### 4.4. Radiological and Dosimetric Properties

To assess the radio-equivalence of gadolinium-infused dosimeters relative to PAGAT (a water-equivalent material), the study followed formulations previously used for the characterization of other gel dosimeters [42,43]. Based on the stoichiometry corresponding to each evaluated gadolinium concentration, the following parameters were calculated: mass density, effective atomic number for photon energy absorption (Z_PEAeff_)—which more appropriately describes the radiological properties in terms of absorbed energy for dosimetric materials—and mass energy–absorption coefficient (µ_en_/ρ). These calculations were performed for the most common effective energies of clinical radiotherapy equipment: 1.6–1.75 MeV for accelerators with a nominal energy of 6 MV [44], 3.8, and 5.0 MeV for those with a nominal energy of 15 and 18 MV [45].

The effective atomic number for photon energy absorption was calculated using the mass energy absorption coefficient through the additive law, following the formulation by Shuvaramu et al. [46]. The (µ_en_/ρ) value was obtained from the tables of Hubbell and Seltzer (1995) [47].

## Figures and Tables

**Table 1:** Pre-irradiation absorbance values of PAGAT and PAGAT infused with Dotarem at 5, 10, and 20 mg/mL, including corresponding standard deviations.

Compounds	Absorbance (a.u.)	SD
PAGAT	0.060	0.003
Omniscan	0.005	0.001
Dotarem	0.007	0.001
Gadovist	0.007	0.001
Omniscan 5 mg/mL	0.061	0.005
Omniscan 10 mg/mL	0.055	0.006
Omniscan 20 mg/mL	0.072	0.003
Dotarem 5 mg/mL	0.069	0.004
Dotarem 10 mg/mL	0.108	0.023
Dotarem 20 mg/mL	1.455	0.136
Gadovist 5 mg/mL	0.063	0.005
Gadovist 10 mg/mL	0.062	0.009
Gadovist 20 mg/mL	0.051	0.005
Gadovist 30 mg/mL	0.040	0.005
Gadovist 40 mg/mL	0.035	0.003

**Table 2:** Dosimetric results for each compound, including the dose threshold and the slope of the dose–response curve, along with their respective standard errors and *p*-values obtained through Student’s *t*-test in comparison with the reference compound (PAGAT). The linear dose range considered for the fit and the presence of response saturation, when observed, are also indicated. Statistically significant values are highlighted in bold.

Dosimeter	Threshold (Gy)	*p*-Value Compared to PAGAT	Slope	*p*-Value Compared to PAGAT	Linear Range (Gy)	Saturation (Gy)
PAGAT	2.46 ± 0.13	1.0000	0.153 ± 0.005	1.0000	2–12	12
Omniscan 5 mg/mL	3.50 ± 0.53	0.0692	0.098 ± 0.005	**0.0002**	3–15	--
Omniscan 10 mg/mL	3.35 ± 0.31	**0.0247**	0.101 ± 0.005	**0.0002**	2–15	--
Omniscan 20 mg/mL	1.75 ± 0.33	0.0520	0.087 ± 0.059	0.1915	2–8	8
Dotarem 5 mg/mL	3.40 ± 0.42	0.0501	0.186 ± 0.008	**0.0065**	4–12	12
Dotarem 10 mg/mL	0.64 ± 0.32	**0.0450**	0.171 ± 0.010	0.0701	1–10	10
Dotarem 20 mg/mL	0.01 ± 0.01	**0.0009**	0.121 ± 0.0004	**0.0077**	0–3	--
Gadovist 5 mg/mL	4.04 ± 0.46	**0.0206**	0.163 ± 0.008	0.1539	4–12	12
Gadovist 10 mg/mL	2.72 ± 0.07	0.0538	0.162 ± 0.003	0.0687	2–10	10
Gadovist 20 mg/mL	1.55 ± 0.12	**0.0009**	0.147 ± 0.004	0.1833	1–10	10
Gadovist 30 mg/mL	1.60 ± 0.11	**0.0011**	0.153 ± 0.004	1.0000	2–8	8
Gadovist 40 mg/mL	1.01 ± 0.17	**0.0004**	0.122 ± 0.005	**0.0016**	1–8	8

**Table 3:** Mass density, effective atomic number for photon energy absorption (Z_PEAeff_), and μ_en_/ρ of Gd-PAGAT and PAGAT dosimeters at radiotherapy effective energies of 1.60–1.75 MeV, 3.8 MeV, and 5.0 MeV.

Dosimeters	Density g/cm^3^	Z_PEAeff_ 1.60–1.75 MeV	Z_PEAeff_ 3.8 MeV	Z_PEAeff_ 5.0 MeV	μ_en_/ρ 1.60–1.75 MeV (cm^2^/g)	μ_en_/ρ 3.8 MeV (cm^2^/g)	μ_en_/ρ 5.0 MeV (cm^2^/g)
PAGAT	1.030	3.38	3.46	3.53	2.742 × 10^−2^	2.098 × 10^−2^	1.905 × 10^−2^
Omniscan 5 mg/mL	1.038	3.42	3.50	3.57	2.737 × 10^−2^	2.097 × 10^−2^	1.906 × 10^−2^
Gadovist 5 mg/mL	1.039	3.40	3.49	3.56	2.738 × 10^−2^	2.097 × 10^−2^	1.906 × 10^−2^
Dotarem 5 mg/mL	1.040	3.40	3.49	3.56	2.737 × 10^−2^	2.097 × 10^−2^	1.906 × 10^−2^
Omniscan 10 mg/mL	1.046	3.43	3.52	3.59	2.733 × 10^−2^	2.096 × 10^−2^	1.907 × 10^−2^
Gadovist 10 mg/mL	1.046	3.42	3.51	3.58	2.735 × 10^−2^	2.097 × 10^−2^	1.907 × 10^−2^
Dotarem 10 mg/mL	1.049	3.43	3.52	3.59	2.733 × 10^−2^	2.096 × 10^−2^	1.907 × 10^−2^
Omniscan 20 mg/mL	1.063	3.46	3.56	3.64	2.727 × 10^−2^	2.097 × 10^−2^	1.911 × 10^−2^
Gadovist 20 mg/mL	1.064	3.46	3.55	3.63	2.728 × 10^−2^	2.097 × 10^−2^	1.909 × 10^−2^
Dotarem 20 mg/mL	1.069	3.46	3.56	3.64	2.727 × 10^−2^	2.097 × 10^−2^	1.911 × 10^−2^
Gadovist 30 mg/mL	1.080	3.48	3.58	3.67	2.723 × 10^−2^	2.097 × 10^−2^	1.913 × 10^−2^
Gadovist 40 mg/mL	1.100	3.52	3.63	3.72	2.716 × 10^−2^	2.096 × 10^−2^	1.915 × 10^−2^

**Table 4:** Percentage values of gelatin, water, BIS, acrylamide, and gadolinium used for each concentration of each contrast agent. THPC is not shown in the table since a concentration of 10 mM was used in all cases.

	Gelatin (% *w*/*w*)	Water (% *w*/*w*)	BIS (% *w*/*w*)	Acrylamide (% *w*/*w*)	Gd (% *w*/*w*)
PAGAT	5.0	88.7	3.0	3.0	0.0
Omniscan 5 mg/mL	5.0	80.7	3.0	3.0	8.1
Omniscan 10 mg/mL	5.0	74.0	3.0	3.0	14.7
Omniscan 20 mg/mL	3.9	62.0	2.3	2.3	29.2
Gadovist 5 mg/mL	5.0	84.2	3.0	3.0	4.5
Gadovist 10 mg/mL	5.0	81.3	3.0	3.0	7.4
Gadovist 20 mg/mL	5.0	73.6	3.0	3.0	15.1
Gadovist 30 mg/mL	3.9	69.3	2.3	2.3	22.0
Gadovist 40 mg/mL	3.9	61.9	2.3	2.3	29.3
Dotarem 5 mg/mL	5.0	81.3	3.0	3.0	7.5
Dotarem 10 mg/mL	5.0	73.9	3.0	3.0	14.9
Dotarem 20 mg/mL	3.9	61.6	2.3	2.3	29.7

## Data Availability

Data are contained within the article.
